# Reversing the Inflammatory Process—25 Years of Tumor Necrosis Factor-α Inhibitors

**DOI:** 10.3390/jcm12155039

**Published:** 2023-07-31

**Authors:** Katharina N. Muth, Juergen Rech, Florian O. Losch, André Hoerning

**Affiliations:** 1 Medical Affairs, Hexal AG, 83607 Holzkirchen, Germany; 2 Department of Internal Medicine III, Rheumatology and Immunology, Friedrich-Alexander-University Erlangen-Nürnberg and Universitätsklinikum Erlangen, 91054 Erlangen, Germany; 3 Deutsches Zentrum Immuntherapie, Friedrich-Alexander-University Erlangen-Nürnberg and Universitätsklinikum Erlangen, 91054 Erlangen, Germany; 4 Clinic for Children and Adolescent Medicine, Friedrich-Alexander-University Erlangen-Nürnberg and Universitätsklinikum Erlangen, 91054 Erlangen, Germany

**Keywords:** cytokines, immune-mediated inflammatory diseases, inflammation, liver, rheumatoid arthritis, tumor necrosis factor alpha-inhibitors

## Abstract

Immune-mediated inflammatory diseases, such as rheumatoid arthritis, psoriatic arthritis, peripheral and/or axial spondyloarthritis, Crohn’s disease, and ulcerative colitis, are characterized by molecular and cellular changes in the immune system. Due to the systemic nature of these diseases, organs such as the liver or cardiovascular system are often affected by the inflammatory process. Tumor necrosis factor-α inhibitor therapy reduces the activation of pro-inflammatory signaling cascades, mitigates the chronic inflammatory process by restoring cellular balance, and alleviates clinical consequences, such as pain and tissue damage.

## 1. Introduction

Inflammation is a regenerative response of the body to mechanical, thermal, chemical, or radiation stimuli [1]. Trauma and certain diseases, such as cancer, infections, diabetes mellitus, or injuries, can also cause inflammation [2,3,4,5,6].

The interaction of various external or internal factors, such as hormonal or genetic changes, can promote the development of an immune disease in the case of a predisposition. However, while inflammation is generally beneficial, it can also cause damage to the organ itself, especially if it is excessive, directed against healthy tissue, or becomes chronic [2]. Such chronic inflammatory processes can manifest in many organ systems as autoinflammatory or autoimmune diseases [6]. The immune system, as a mediator of the inflammatory response, is constantly excessively active, even in the absence of possible trigger factors. Often, the origin of this excessive reaction is the misdirected activity of cells of the innate immune system, such as dendritic cells or macrophages [7]. If the stimulatory properties of these cells prevail over the long term, dendritic cells, for example, can promote the activation of T cells of the adaptive immune system, thereby driving inflammation and, thus, contributing to the development of autoimmune diseases [7]. These long-lasting, unregulated inflammatory processes can eventually lead to impaired self-tolerance of certain body structures by causing misdirected activity of the B cells of the adaptive immune system, which are responsible for the formation of characteristic autoantibodies against body structures, such as in systemic lupus erythematosus [6].

Cytokines are part of the non-specific (innate) and the specific (acquired) immune defense. They are a broad group of signaling proteins through which immune cells communicate with each other and can, thus, stimulate or inhibit immune responses. Cytokines are produced transiently and act as humoral regulators activating or deactivating cells and protecting tissues from damage [8]. The imbalance between pro- and anti-inflammatory cytokine levels can lead to significant negative health impacts. Pro-inflammatory cytokines ensure the attraction of immune cells to the site of infection, increased blood flow to the affected tissue, and the activation of immune cells. Anti-inflammatory cytokines, on the other hand, ensure that the inflammation subsides, and the activated cells become controlled again. In addition, the interaction of pro- and anti-inflammatory cytokines regulates the effective course of the immune response. The balance of pro-inflammatory and anti-inflammatory cytokines is a prerequisite for an immune defense to run smoothly, but also for the immune reaction to come to a standstill again. If the balance and, thus, the interplay of pro- and anti-inflammatory cytokines is disturbed, serious illnesses occur because either the immune defense is insufficient, or the immune reaction is excessive [9]. For example, if pro-inflammatory cytokines are present in excess or if too few anti-inflammatory cytokines are present, chronic inflammation will result.

## 2. Tumor Necrosis Factor-α—Central Downstream Mediator of Chronic Inflammatory Response

**Figure 1:**
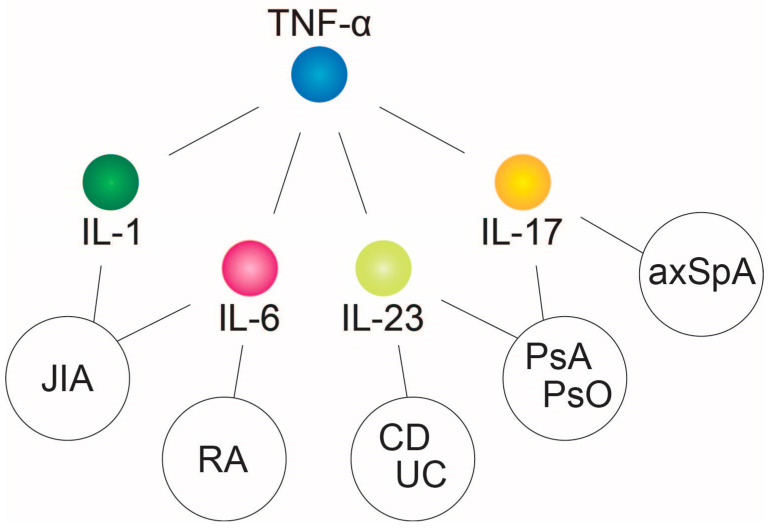
The cytokine TNF-α is a superordinate and crucial node within the hierarchical structure of cytokine networks underlying various chronic inflammatory diseases. Abbreviations: axSpA, axial spondyloarthritis; CD, Crohn’s disease; IL, interleukin; JIA, juvenile idiopathic arthritis; PsA, psoriatic arthritis; PsO, psoriasis; RA, rheumatoid arthritis; TNF, tumor necrosis factor; UC, ulcerative colitis.

The cytokine tumor necrosis factor (TNF)-α plays a central role in the pathogenic inflammatory process. It is also recognized as a superordinate, critical hub within the hierarchical structure of cytokine networks underlying various chronic inflammatory diseases (Figure 1). Other pro-inflammatory cytokines, such as interleukin (IL)-1, IL-6, and IL-17/23, only influence subordinate networks in the inflammatory process [10,11].

During inflammation, TNF-α is released by macrophages, dendritic, and lymphocytic cells. It is also secreted by mast cells, nerve cells, endothelial cells, lipocytes, heart muscle cells, fibroblasts, and lymphocytes. TNF-α triggers apoptosis, cell proliferation, and cell differentiation, but also the release of other cytokines. In the chronic inflammatory state, TNF-α shifts the predominant cytokine environment towards pro-inflammatory cytokines, such as IL-1, IL-6, IL-17, IL-22, and interferon gamma (IFN-γ), while simultaneously reducing the secretion of anti-inflammatory cytokines, in particular IL-4 and IL-10 [12,13].

Together with IL-12 and IL-18 or transforming tumor necrosis growth factor (TGF)-β, IL-6, and IL-23, TNF-α induces the differentiation of naïve CD4+ T cells into pro-inflammatory T helper cells of type 1 (T_H_1) and type 17 (T_H_17), respectively [6,12,13]. T_H_1 cells are characterized by, among other things, the expression of the chemokine receptor CXCR3 (CXCR3+CCR6-) and the release of IFN-γ, TNF-α, and IL-2 when combating pathogens. T_H_17 cells are characterized by the high surface expression of the chemokine receptor CCR6 (CCR4+CCR6+), and occur mainly in the mucosa of the intestine, skin, and lungs. Here, they are mainly involved in the defense against extracellular bacteria and fungi [14]. Depending on the cytokine environment present, the T_H_17 cells differentiate into different phenotypes: in a healthy state, differentiation is predominantly into an infection-regulating T_H_17 cell type, but in chronic inflammation, cells of the pathogenic T_H_17.1 subtype are mainly formed, whose number correlates with disease activity in both rheumatoid arthritis (RA) and Crohn’s disease (CD) [13,14,15,16,17].

In contrast to the partly anti-inflammatory T_H_17 cells, the excessive pathogenic T_H_17.1 cell population is characterized by a high proliferation rate, as well as resistance to glucocorticoids and the suppressing effects of regulatory T cells (T_reg_) [13]. T_H_17.1 cells also differ from T_H_17 cells in terms of their secreted cytokine patterns. The pathogenic T_H_17.1 cells, primarily present in RA and CD, secrete only small amounts of IL-17A and IL-17F, which is why monotherapy with an IL-17A antibody shows only moderate effects in these clinical conditions [13]. Instead, they release high concentrations of TNF-α, IFN-γ, and granulocyte-macrophage colony-stimulating factor (GM-CSF) [13,14]. GM-CSF can subsequently stimulate macrophages to release more IL-1β, TNF-α, and IL-6, and, together with IL-23 released from T_H_17 cells, further promote the inflammatory cycle [13].

**Figure 2:**
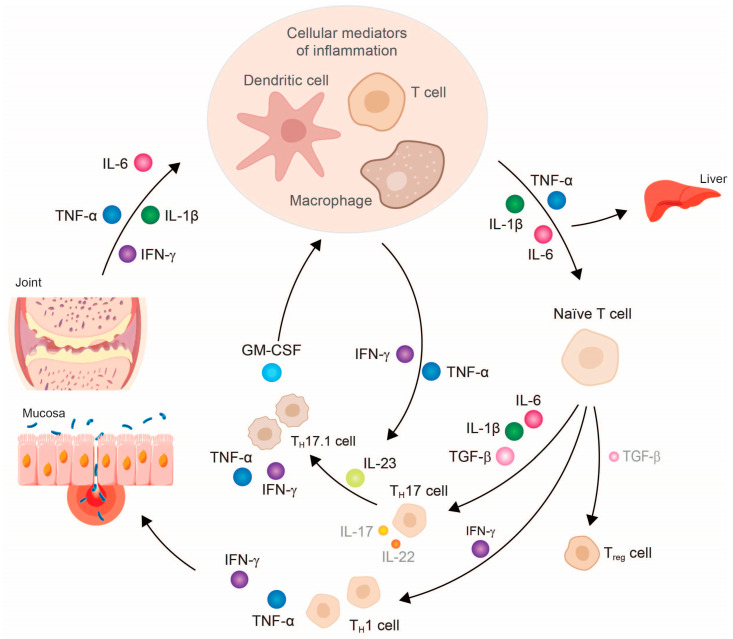
Simplified presentation of the development and chronification of inflammatory processes by the impaired balance between pro- and anti-inflammatory cytokines, e.g., in rheumatoid arthritis. Abbreviations: GM-CSF, granulocyte-macrophage colony-stimulating factor; IFN, interferon; IL, interleukin; TGF, tumor necrosis growth factor; T_H_, T helper cell; TNF, tumor necrosis factor; T_reg_, regulatory T cell. The size of the cytokines depicted schematically represents their released amount.

Due to this disturbed cytokine milieu and the resulting chronic inflammatory event, patients with RA or axial spondyloarthritis may experience progressive tissue and joint damage (Figure 2). The infiltration of inflammatory cells—for example, granulocytes, macrophages, and lymphocytes—into the affected tissue, such as the synovium in RA or the colon in CD, is characteristic of the local reaction [15,16,17]. The production and secretion of chemokines responsible for the migration and accumulation of these inflammatory cells at the site of inflammation also appear to be stimulated by TNF-α [15,16].

However, this altered cytokine environment also influences the function of organs not primarily affected by the inflammatory processes, for example, the liver. Metabolizing enzymes can be inhibited in the liver and thereby influence the pharmacokinetics of the substances and drugs metabolized via these enzymes [18,19,20,21]. The cause is a restriction of the activity of cytochrome P450 CYP3A4 in the CYP enzyme system in the liver induced by pro-inflammatory cytokines (e.g., IL-6, TNF-α, and IL-1Ra), an effect that is stronger at high concentrations of these cytokines (i.e., during highly perturbated and excessive inflammatory events) [18,19,20,21]. These inflammatory processes or the pro-inflammatory cytokines cause transcriptional, post-transcriptional, and epigenetic changes in the expression of the metabolizing enzymes as well as transport proteins of the liver, and, thereby, influence the plasma level, bioavailability and, thus, efficacy of some drugs [18,20].

In addition, TNF-α mediates hepatocellular apoptosis and appears to be involved in sterile intrahepatic inflammation [22]. The constant, systemic inflammatory process and high concentrations of TNF-α may contribute to the progression of chronic liver inflammation in non-alcoholic fatty liver disease (NAFLD). Here, TNF-α leads to the accumulation of collagen and can lead to the remodeling of the matrix, resulting in fibrosis through the induction of apoptosis [23,24]. In the liver, TNF-α not only affects the increased release of pro-inflammatory cytokines, such as IL-6 and acute-phase proteins, but it may also lead to an increase in insulin resistance by the phosphorylation of insulin receptors, as well as to anemia, through the induction of hepcidin in inflammatory bowel disease (IBD) [22,25,26].

Furthermore, chronic inflammatory processes, and specifically the concentration of TNF-α, drive cardiovascular events, such as atherosclerosis, atherothrombotic disease, and venous thromboembolism [27]. The biomarkers associated with an increased risk of cardiovascular events include fibrinogen and endothelial dysfunction, as well as the cytokines IFN-γ and TNF-α, and these two inflammatory markers have also been shown to be predictive of future cardiovascular events [28].

## 3. TNF-α Inhibitor Therapy Restores Cytokine Balance and Normalizes Organ Function

**Figure 3:**
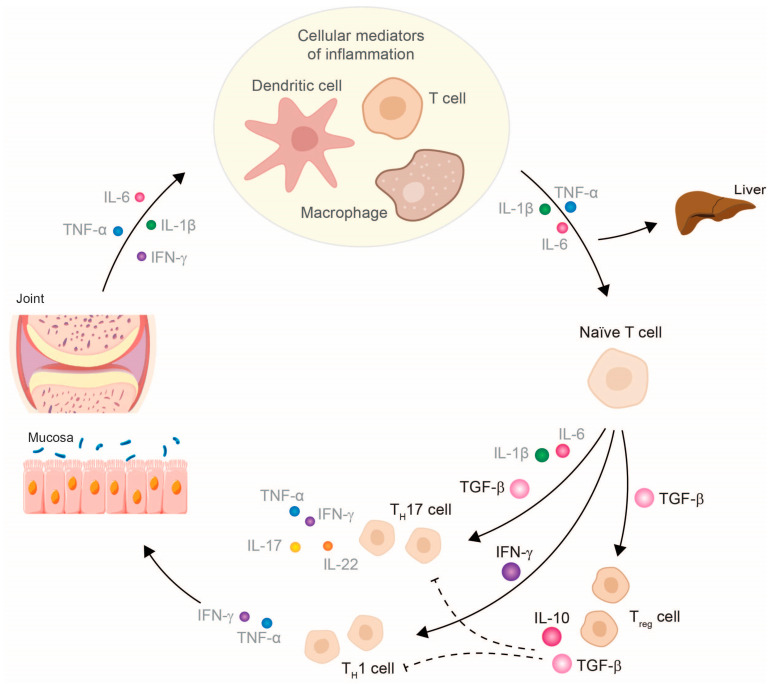
Therapy with a TNF-α inhibitor restores the cytokine balance in chronic inflammatory diseases and, thus, also the cellular balance. Abbreviations: IFN, interferon; IL, interleukin; TGF, tumor necrosis growth factor; T_H_, T helper cell; TNF, tumor necrosis factor; T_reg_, regulatory T cell. The size of the cytokines depicted schematically represents their released amount.

TNF-α inhibitors can counteract the entire disease process in patients with chronic inflammatory diseases (e.g., RA, psoriatic arthritis (PsA), CD, and ulcerative colitis (UC)) by fundamentally modulating the molecular and cellular changes of several inflammatory networks of the innate immune system [6,10,16,17,29,30,31]. The focus is on the reduction of TNF-α due to TNF-α inhibitors and the subsequent pro-inflammatory cytokines, such as IL-1, IL-6, or IFN-γ [16,29,30,31], and, thus, the restoration of cellular homeostasis. The decrease in these pro-inflammatory cytokines leads to the normalized differentiation of T cell subtypes, which increases the number of anti-inflammatory cells (e.g., T_reg_ cells and anti-inflammatory T_H_17 cells) and reduces the number of pathogenic T_H_17.1 cells [13,14,16]. T_reg_ cells can now exert their suppressing function by releasing the anti-inflammatory cytokines IL-10 and TGF-β, which subsequently influence cytokine release by effector T cells (Figure 3). In IBD patients, it has been shown that therapy with a TNF-α inhibitor can also exert a modulating effect on specific subtypes of B lymphocytes (CD24hiCD38hi-B cells). The inhibition of TNF-α leads not only to a numeric reconstitution of B cellular subset distribution, but also achieves a normalization of IL-10 production. This observation reflects at least a reconstitution of the immune system [32]. Further studies have shown that a restoration of B cell numbers correlated with the biological response to the TNF-α inhibitor therapy [33,34]. Regarding the reconstituting effect, earlier studies have provided evidence that infliximab exerts its anti-inflammatory effects by modifying the IBD-characteristic cytokine networks. Ringheanu and colleagues found that infliximab downregulated the production of inflammatory cytokines by showing that monocytes produced less TNF-α, IL-1β, IL-6, and IL-8 mRNA, as measured at the RNA and protein level [35]. In addition, activated T cells isolated from colonic biopsies of patients with CD cultured in the presence of infliximab were found to decrease their expression of IFN-γ [36].

Further findings indicate that in IBD, TNF-α blockage has been shown to result in reduced intestinal permeability through decreased endothelial cell apoptosis and decreased permeability of tight junctions, increased Treg cell activity, reduced activity of various inflammatory mediators and T cells, and a reduction in inflammation-mediated mucosal angiogenesis by preventing the production of vascular endothelial growth factor A from intestinal fibroblasts [37,38].

Following the treatment of RA patients with infliximab, a restored population of Treg cells expressing low levels of CD62L was identified that mediates the suppression of T effector cells via TGF-β and IL-10, resulting in reduced IFN-γ production [39]. Patients clinically responding to the fully humanized anti-TNF-α antibody adalimumab also showed an increased percentage of FoxP3+ cells with restored regulatory function, as these cells suppress and resist conversion to T_H_17 cells [40].

Studies investigating the effects of anti-TNF-α therapy for B cell subsets in RA are scarce and yielded, in part, similar results when compared to the IBD setting. The frequency and the absolute number of mature/memory (CD24hiCD27+) cells, of immature (CD24hiCD38hi) transitional B cells, and of IL-10-producing Breg cells have been shown to be decreased in RA versus healthy controls, and increased after therapy with MTX together with the anti-TNF-α inhibitors, adalimumab or etanercept [41,42,43,44].

Consequently, the inhibition of TNF-α attenuates inflammatory reactions and allows anti-inflammatory signaling pathways to prevail again, giving the chance for a restoration of the homeostatic immunological balance [13,14,16,29,30,45]. TNF-α inhibitors also reduce the expression of molecules involved in chemotaxis, invasion, and the adhesion of immune cells at the site of inflammation, thereby reducing local inflammatory processes [12,13,30].

Therapy with TNF-α inhibitors can thus be used to break the vicious cycle of chronic inflammation early and effectively [46,47], since TNF-α blockade weakens several inflammatory networks and promotes anti-inflammatory processes [13,14,29,30,31,32,48]. Consequently, the blockade of TNF-α has a systemic effect and is not restricted to a specific inflammatory disease, which is why the development of further organic manifestations can be prevented and chronic comorbidities can be treated efficiently [6,10,11,49,50,51]. In contrast, the therapeutic inhibition of other pro-inflammatory cytokines, such as IL-1, IL-6, or IL-17/23, only affects downstream networks and, therefore, has a narrowly defined mode of action in the inflammatory process [10].

In the case of the liver, therapy with a TNF-α inhibitor can thus be associated with the normalization of liver function and lead to an improvement in hepatic cytochrome activity, since lower concentrations of inhibitory cytokines, such as IL-6 and IL-1Ra, are present [18,19,20,21,22,23,24,30]. In addition, TNF-α inhibitors cause a reduction in acute-phase proteins, such as fibrinogen, haptoglobin, hepcidin, and serum amyloid A in the liver [25,26,50].

Therapy with TNF-α inhibitors can reduce the risk of overall cardiovascular events, myocardial infarction, and serious venous thrombotic events (VTE) in RA patients [52]. The normalization of fibrinogen and thrombocytosis and the reduction of the expression of vascular endothelial growth factor, as well as of oxidative stress and endothelial dysfunction, by TNF-α inhibitors may reduce the relevant risk factors for atherosclerosis and cardiovascular events in patients with chronic inflammatory disease and increased cardiovascular risk, such as in RA [25,45,52,53,54]. Patients with IBD are at increased risk of thrombotic events. Thus, the epidemiological aspects and drug-related risks of venous (VTE) and arterial thrombotic events (ATE) were summarized in the *Evidence-Based Guideline* in order to provide recommendations for the optimized medical treatment of IBD patients. The *Evidence-Based Guideline* concluded that TNF-α inhibitors are associated with a decreased risk of VTE and arterial events, implicating a potentially protective role of TNF-α inhibitors against VTE in IBD patients [55]. In this regard, csDMARDs and JAK-inhibitors indicated for the treatment of chronic inflammatory diseases are associated with increased risks of cardiovascular events and VTE in comparison to TNF-α inhibitors [27,56].

CD is also associated with an increased risk of osteoporosis. A retrospective analysis suspected that TNF-α inhibitor therapy in post-menopausal/estrogen-deficient patients could prevent osteoporosis but further studies are needed for clarification of the impact of TNF-α inhibitors [57]. Therefore, organ functions and the general clinical condition of the patient improve due to the balance of pro-inflammatory and anti-inflammatory factors restored by therapy with a TNF-α inhibitor [21,22,25,26]. Notably, the comorbidities of patients with chronic inflammatory diseases need to be considered in treatment decision-making. Expert recommendations for the detection and monitoring of comorbidities in the treatment of psoriasis, PsA, and RA patients were developed to provide further guidance on achieving improved disease outcomes [58,59].

## 4. TNF-α Inhibitor Therapy and Its Clinical Impact

With infliximab as the first biologic agent approved for the therapy of CD in 1998, TNF-α inhibitors have a long history regarding the treatment of chronic inflammatory diseases. By gaining approval in increasing numbers of indications, TNF-α inhibitors soon became an attractive treatment option for numerous chronic inflammatory diseases. TNF-α is involved at each stage of the pathogenesis of autoinflammation through its control of multiple cytokine networks and their activity. Hence, the blockade of TNF-α crucially results in the omission of subsequent pro-inflammatory signaling pathways regulated by subordinate cytokines. TNF-α inhibitors interrupt the cycle of inflammation early and effectively, thus, preventing the formation of comorbidities. For example, the development of extra-intestinal manifestations in pediatric CD patients could be significantly reduced, or even prevented, by the early use of infliximab [49,50,60].

Currently, five different TNF-α inhibitors of chronic inflammatory diseases are available: infliximab, etanercept, adalimumab, certolizumab pegol, and golimumab. Although all TNF-α inhibitors are designed to bind TNF-α, they have different molecular structures, which translate into different pharmacokinetic properties, binding affinities, as well as dosage regimens (Supplementary Table S1) [61]. Precisely, infliximab, adalimumab, and golimumab are anti-TNF-α monoclonal antibodies (mAbs), whereas certolizumab pegol is a pegylated single Fab’ antibody fragment and etanercept is a fusion protein, in which TNF receptor 2 is fused to the human IgG1-Fc domain. Adalimumab has the broadest spectrum of therapeutic approvals, ranging from uveitis and IBD to hidradenitis suppurativa; thus, patients with chronic inflammation manifested in various organ systems, such as PsA patients, could be treated with only one drug. Here, adalimumab demonstrates efficacy in several domains, i.e. peripheral arthritis, axial involvement, enthesitis, and dactylitis, as well as extra-articular manifestations [62]. 

Because TNF-α inhibitors have been on the market for a long time, their safety profiles for vulnerable patient populations, such as pediatric patients, can also be extensively established. Importantly, as chronic diseases mainly last for the whole lifetime, the effect of TNF-α treatment on the further course of the disease, and also with regard to the total health of pediatric patients, is of great relevance. It is therefore worth mentioning that TNF-α inhibitors are currently the only biologic drug approved for the anti-inflammatory therapy of pediatric patients suffering from IBD.

Furthermore, the early application of biologic agents significantly prevents treatment failure in pediatric IBD [49]. In 2020, a randomized trial directly comparing first-line infliximab with exclusive enteral nutrition or corticosteroids as a first-line treatment in pediatric patients with moderate-to-severe CD was provided by Jongsma and colleagues [46]. Of the patients treated with first-line anti-TNF-α therapy, a significantly higher percentage achieved clinical and endoscopic remission. In addition, first-line TNF-α-blocker therapy needed less dose escalation while achieving mucosal healing [46]. In addition, the probability of continued clinical remission at week 52 with azathioprine monotherapy was higher in children who received infliximab as a first-line remission-inducing therapy. Comparable results were shown for adalimumab [47].

TNF-α inhibitors have long been established for the treatment of JIA, and data for treatment duration of more than 10 years is available confirming its effectiveness and tolerability in children and adolescents [63]. This understanding and practical experience, especially for the treatment of children, is missing for other biological treatments that have recently been developed and approved, such as Janus kinase (JAK) inhibitors, where data collection on efficacy and safety is still ongoing.

Therapeutic strategies such as “hit hard and early” and “treat-to-target”, especially with TNF-α inhibitors, can modulate the course of the disease enormously, as they have been shown to lead to drug-free remission, lower disease activity, and higher functional status, which ultimately leads to an improvement in patients’ quality of life [64,65,66].

In CD, the early and effective use of TNF-α inhibitors has also been shown to prevent the development of disease complications, for example, strictures or penetrating ulcerations and disease progression, which led to a lower risk of surgery during disease progression and significantly reduced the risk of penetrating complications, but not stenosing complications [60].

The current study data provide evidence that treating patients with inflammatory diseases, such as CD or juvenile idiopathic arthritis (JIA), early after diagnosis with TNF-α inhibitors prevents not only disease progression, but also the loss of structural functioning and other disease complications [60,67]. Especially in young patients, an effective therapy with rapid onset is necessary to prevent growth retardation and structural damage. For pediatric patients with IBD, data from the *RISK* study demonstrates that patients treated early with TNF-α blockers regain weight and body growth is facilitated [51]. TNF-α inhibitors were shown to induce disease remission within three months, thus, being superior to other immunomodulators [68].

This concept of a “window of opportunity”, meaning that early treatment with TNF-α inhibitors can impact the further disease course, is also established in adult patients suffering from RA, PsA, and CD [69]. In PsA, TNF-α inhibitor treatment can decrease the rate of radiographic progression, thus, reducing structural damage, such as erosion and joint space narrowing [66]. The preservation of structural functions and, consequently, of physical activities is associated with a better quality of life of patients treated with TNF-α inhibitors, as reported by real-world data from PsA patients [65]. In this non-interventional study, TNF-α inhibitors resulted in significant improvements in disease activity indicators, i.e., DAS28-CRP and DAPSA28; in patient-reported outcomes; as well as in the improved working ability of patients [65]. This effect on structural preservation was also shown for etanercept in early RA, where it not only slowed the rate, but even halted the radiographic progression of the disease in most of the patients [65]. The prevention of the further functional disability of the patients allows them to participate in social life and to work, leading to improved well-being and quality of life. Consistently, early therapeutical intervention with biologics, i.e., TNF-α inhibitors, is supported by national and international guidelines for the treatment of patients with moderate-to-severe disabling CD. In a retrospective study, this therapy regimen was shown to positively affect the disease course as more patients achieved endoscopic remission while observing a reduced number of structural behavior issues and surgeries [67]. Thus, TNF-α inhibitors also indirectly contribute to cost savings, as fewer complications and lower hospitalization rates also reduce the burden on the healthcare system [70]. Currently, therapeutic drug monitoring (TDM) is being debated in terms of the management of TNF-α inhibitor therapy for inflammatory diseases, such as RA or IBD. It enables the physician to proactively adapt the therapy algorithm if the patient experiences no clinical response. This precise and individual therapy adjustment can also lead to cost savings in the long term, as the exact amount of biologics can be determined with the avoidance of overtreatment [71].

At present, research is being performed in order to find the most suitable treatment options for the individual patient in clinical practice. As direct head-to-head studies are scarce, several network meta-analyses have compared the various agents approved for their therapeutic utility in treating several inflammatory diseases, i.e., IBD, ankylosing spondylitis (AS), RA, and PsA.

TNF-α inhibitors have proved that they continue to have an enormous relevance in the therapeutic landscape and are still established as therapeutics to control chronic inflammatory diseases despite, the era of newly developed types of treatment. This becomes evident when the different mechanisms of action are examined and compared with regard to their safety profiles and effectiveness.

The observational cohort *JAK-pot* study analyzed the real world data of four classes (JAK-inhibitors, TNF-α inhibitors, IL-6 inhibitor, abatacept) of second-line treatment approved for RA [72]. Their effectiveness was compared by evaluating the time of and reasoning for drug discontinuation, as well as Clinical Disease Activity Index (CDAI) response rates at 1 year. The key finding was the comparable clinical effectiveness among those four therapeutic agent groups. However, there were differences in the reasons for drug discontinuation, as JAK inhibitors were mainly stopped for safety reasons and less commonly as a result of their ineffectiveness.

As such, a recent study highlighted the importance of TNF-α inhibitors in AS therapy, showing that TNF-α inhibitors were superior to IL-6, -17, -23, and JAK inhibitors regarding efficacy and safety and should be used as the preferred replacement for conventional drug therapy in patients suffering from a rapid disease progression and physical functional limitations [73]. For the treatment of radiographic axSpA (r-axSpA), a direct comparison of the TNF-α blocker adalimumab with the IL-17 inhibitor secukinumab revealed that both biologic agents were comparably efficient in reducing radiographic progression over 2 years [74].

Comparative studies on therapies for IBD also reflect these results, confirming the importance of TNF-α inhibitors for the treatment of inflammatory diseases. In a post-hoc analysis of pivotal clinical trials, the efficacy of biologic agents was investigated for achieving endoscopic healing in CD. The comparison revealed that both tested TNF-α blockers (adalimumab and infliximab) were superior to vedolizumab and ustekinumab for achieving endoscopic healing of the ileum and colon in patients with CD [75]. Consistent with this, a systematic review and meta-analysis evaluating the efficacy and safety of infliximab and vedolizumab in adult patients with moderate-to-severe CD or UC showed that infliximab has significantly better efficacy in the induction phase and comparable efficacy during the maintenance phase [68].

Moreover, the neutralization of a single pro-inflammatory cytokine, such as TNF-α, can directly lead to the improvement of symptoms, such as pain. Pain is of enormous relevance to people with RA. TNF-α has previously been regarded as a mediator of pain due to its involvement in mechanical hyperalgesia after injury [76]. Hess and colleagues have recently demonstrated that both mice with TNF-α-mediated arthritis and humans with RA show enhanced brain activity in the centers involved in pain perception and the control of emotions [77]. TNF-α inhibitor treatment reverses this enhanced central nervous system activity in both mice and humans within 24 h, again, even before the clinical effects on arthritis are observed. In addition, they were also able to show that the rapid relief of abdominal pain may be explained by altered pain perception in the brain of CD patients owing to the inhibition of TNF-α [78].

Cavanagh and colleagues were able to show that the pathologically increased neuronal activity caused by peripheral inflammation in the brain can be rapidly downregulated by TNF-α inhibitors. This is underpinned by positron emission tomography studies. There is also evidence that TNF-α inhibitors alter serotonin transporter expression in the brain [79].

The fact that TNF-α inhibitors have pain-relieving properties is another important feature in addition to inflammation control and the preservation of structural integrity, thus leading to a direct improvement in quality of life in patients.

Over the years, TNF-α inhibitors’ safety profile has also been established during pregnancy and lactation. Based on the currently available study data from IBD and on rheumatic indications, TNF-α inhibitors seem to be safe and effective without a significant maternal or fetal risk (i.e., no increase in the frequency of miscarriages or congenital anomalies, no difference in growth and development) [61,80,81].

Presumably, this is due to the low placental transfer, i.e., of certolizumab pegol, with suggested comparable safety for etanercept [80].

As a result of immunosuppression, patients undergoing anti-inflammatory therapy are susceptible to infections caused by bacteria, fungi, or viruses. Therapy with TNF-α inhibitors can result in the reactivation of latent infections, such as tuberculosis or the hepatitis B virus. Thus, patients need to be screened for latent infections prior to the initiation of the TNF-α therapy in order to reduce the risk of reactivations [62]. Active tuberculosis or other serious infections, such as sepsis and opportunistic infections, as well as moderate-to-severe heart failure (NYHA-class III/IV), are contraindications for TNF-α inhibitors.

Further, this systemic immunomodulation mediated by TNF-α inhibitors may lead to unpredictable reactions to vaccinations; thus, patients under TNF-α therapy should not be vaccinated with live vaccines.

The long approval history of TNF-α inhibitors has allowed us to gain valuable insights into their long-term application. Clinical data registries are available reporting the long-term effects, which are essential to monitor the further implications or side effects of therapy that may have not become apparent for several years, such as cancer. A recent network meta-analysis assessed the risk of malignancies (such as non-melanoma skin cancer and lymphoma), tuberculosis, and infections in RA patients with different TNF-α therapies. According to their findings, none of the 10 studied anti-TNF therapies resulted in an increased risk of serious infections, malignant tumors, and tuberculosis infection compared to placebo or conventional DMARDs therapy [53]. Further, RA patients with serious infections before the initiation of TNF-α inhibitor therapy were not at increased risk of subsequent serious infection and the hospitalization rate was comparable between patients continuously treated with the same TNF-α inhibitor and patients who switched to another TNF-α inhibitor, or even to a different mode of action [54]. And also, for IBD patients, the risk of melanoma or hepatosplenic T-cell lymphoma was low or even not established [62].

Due to the existing overlap of the involved signaling pathways and disease patterns, it is also possible that not only the initial manifestation influences the development and pathology of a further chronic inflammation, i.e., along the gut-liver, gut-joint, and gut-brain axes, but also that the therapeutic modulation of one disease has an impact on other organ systems [82]. For TNF-α inhibitors, for example, cases of treatment-induced or exacerbations of skin manifestations, such as psoriasis were reported [83]. Further, rare cases of drug-induced hepatotoxicity have been reported for infliximab monotherapy used for the treatment of IBD. However, the reported serologic and pathologic characteristics of this liver injury mostly resolve following the discontinuation of infliximab [84].

## 5. Future Directions

When comparing the effectiveness of TNF-α inhibitors and advanced therapies with other modes of action indicated for chronic inflammatory diseases, practical experience and data on the outcomes of the long-term therapeutical use of recently approved therapies are very limited, thus, data collection on efficacy and safety is needed in order to make an optimal treatment choice. Through years of experience, the safety profile of TNF-α inhibitors is well established and has even been described during pregnancy, breastfeeding, and in pediatric patients. Consequently, treatment guidelines for IBD and rheumatic diseases have been adapted accordingly and include TNF-α inhibitors in their recommendations, making TNF-α inhibitors an important option in the therapeutic landscape.

Therapy with a TNF-α inhibitor has manifold effects: it effectively controls inflammation, can result in the preservation of structures, and it reduces pain [77]. TNF-α inhibitors can reverse the systemic chronic inflammatory process through their ability to modulate disease at the molecular and cellular levels. Together, the alterations of molecular and cellular properties re-establish balanced conditions, eventually re-establishing organ function.

Based on the multiple effects of blocking TNF-α, an earlier use in the therapy algorithm has been discussed in order to benefit from the positive effects of TNF-α therapy at an earlier stage of disease. TNF-α inhibitors, when used early after disease diagnosis, can positively impact the course of disease by preventing structural damage and the development of comorbidities. This is of great relevance for preserving physical function and activities, eventually leading to an increased quality of life for patients. For vulnerable patient populations, for example, pediatric patients needing long-term medical treatment, drug tolerability and safety should be considered in addition to effectively controlling disease activity.

Treatment strategies regarding the early initiation of biologics and their withdrawal upon reaching deep remission, or even discontinuation of a less effective immunomodulator, continue to be discussed in the field of chronic inflammatory diseases. The de-intensification of an anti-TNF-α treatment for patients in long-term remission may represent a goal to achieve in different clinical scenarios and aspects. De-escalation strategies may include a dose reduction or a lengthening of the drug administration intervals while maintaining the same clinical effects. In a recent systematic review, results from 20 TNF-α inhibitor de-escalation studies (14 RCTs and six non-RCTS) determining the actual reduction in the occurrence of adverse events when de-escalating the existing therapy in different inflammatory conditions, such as psoriasis, axSpA, RA, and IBD, were summarized. [85] While the overall quality of evidence in these summarized studies was limited, Bouhuys et al. still described how an anti-TNF-α de-escalation did not reduce the occurrence of infections and skin manifestations compared to patients who continued standard dosing according to the label’s recommendation [85].

The *Rheumatoid Arthritis in Ongoing Remission* (RETRO) study did demonstrate a successful dose reduction or even complete withdrawal of DMARDs in about half of the RA patients, meaning that their status of disease remission could be maintained without a decline in physical function [86,87].

Dose tapering always bears the risk of disease relapse; thus, a tight monitoring of the individual disease activity is required, as reported in a systematic review by Little et al. and also in the meta-analysis by Zhang et al. [88,89]. A recent sophisticated review reported relapse rates in a general range between 40% and 50% over a 2-year period following the discontinuation of the anti-TNF−α treatment. Comparable rates were obtained in tapering studies in axSpA and PsA patients, in whom clinical remission or low disease activity could be achieved in about 50% of cases, but disease flares were often caused [90]. Thus, anti-TNF−α withdrawal may only be considered in low-risk patients, although there are currently no controlled studies existing to support this approach.

Although data on the long-term outcomes of patients with CD after infliximab withdrawal are limited, long-term follow-up results over 7 years from the STORI cohort showed that about one-fifth of the patients did not restart infliximab or another biologic agent, and this did not result in major complications. Of note, 70% of the patients had no failure with the de-escalation strategy, meaning no development of a major complication and no failure of the re-initiation of infliximab therapy [91]. In most of the patients who experienced flares, restarting the biologic treatment resulted again in disease remission, while concomitant immunomodulators may attenuate potential biologic immunogenicity. As mentioned before, the interpretation of these data needs to be conducted carefully, as prospective RCTs are scarce.

Gomes et al. proposed that TDM may support attempts at drug withdrawal by providing guidance for the physician on whether to stop the immunomodulator or the TNF-α inhibitor when a combination therapy is used [92].

In line with the aforementioned meta-analyses, the results of the SPARE trial were recently published by Louis et al. and reported that in CD patients in sustained steroid-free remission undergoing combination therapy with infliximab and an immunomodulating drug, the withdrawal of infliximab should only be considered after a careful assessment of the risks and benefits for the individual patient, whereas the withdrawal of the immunomodulatory drug could generally represent the preferred strategy [93].

Taken together, as there are no guidelines available for the tapering or discontinuation of current treatments of inflammatory diseases, we suggest that tapering DMARDs should, for one, be based on shared decision making with the patient being allowed to find individual ways of reducing the drug burden, as is currently shown for RA patients.

Further, to avoid the worsening of disease activity or quality of life following the drug tapering, a close monitoring of these patients should be conducted, i.e., by employing biomarkers of inflammation, such as fecal calprotectin in the case of IBD to predict a disease relapse after anti-TNF-α withdrawal or de-escalation.

Because of all these reasons, early therapy with TNF-α inhibitors seems to be important for patients with chronic inflammatory diseases. With the introduction of biosimilars, this formerly very expensive therapy has been made affordable, allowing more patients to receive this important treatment. In daily practice, substantial cost savings to the healthcare system are possible if biological-naive patients are initially treated with biosimilars, or if patients who already receive the reference biological DMARDs (bDMARDs) are switched to the corresponding biosimilar product [94]. The real-world economic impact of the use of biosimilars in the therapeutic landscape of inflammatory rheumatic diseases, including RA, AS, and PsA, becomes obvious in the current meta-analysis performed by Kim et al. [95]. In the European economic area market, the use of biosimilars can lead to a cost decrease per treatment day of 13% compared to therapy with the reference product, but with a concomitant increase of 19% in volume per treatment day [95]. The UK can be considered as a pioneer in this area, as the use of biosimilars is highest here. Correspondingly, the NHS has indicated further promotion of therapy with biosimilars in order to save £300 million a year by 2021.

## 6. Conclusions

Despite their “age” and the era of newer modes of action, TNF-α inhibitors continue to be a central and proven component of the therapeutic armamentarium in chronic inflammatory diseases with easily manageable possible side effects. Therapy with a TNF-α inhibitor has manifold effects, thus, making it an inevitable part of the landscape of anti-inflammatory therapies. By modulating molecular and cellular features, TNF-α inhibitors control inflammation by reversing the inflammatory processes. Thus, TNF-α-inhibitor therapy leads to an effective reduction in inflammation, resulting in preserving organic and structural integrity and controlling pain, resulting in a better quality of life for patients.

## Data Availability

Not applicable.

## Supplementary Materials

The following supporting information can be downloaded at: https://www.mdpi.com/article/10.3390/jcm12155039/s1, Table S1: displaying the five approved anti-TNF-α inhibitors in chronological order of first approval by Food and Drug Administration (FDA).
