# Supplementary material for: Reversing the Inflammatory Process—25 Years of Tumor Necrosis Factor-α Inhibitors
Source: J Clin Med. 2023 Jul 31;12(15):5039. doi: 10.3390/jcm12155039 (PMC10419406; doi:10.3390/jcm12155039)
Supplement: Supplementary file 1 [file jcm-12-05039-s001.zip › jcm-2443756-supplementary.pdf]

# Supplementary

**Table S1.** displaying the five approved anti-TNF-$\alpha$ inhibitors in chronological order of first approval by Food and Drug Administration (FDA).

| TNFa inhibitor | First Approval date by FDA | Indications | Administration Route, Dosing | Comments |
|---|---|---|---|---|
| Infliximab | 1998 (CD,FDA) | CD and UC (adult and pediatric) RA, AS, PsA, Psoriasis | iv, 3 to 5 mg/kg at 0, 2, 6 weeks, then every 6-8 weeks | Pediatric IBD |
| Etanercept | 1998 (RA, FDA) | JIA, RA, AS, nr-axSpa, PsA, Ps (adult and pediatric) | sc, 25 mg twice a week or 50 mg/week | Monotherapy possible (no co-treatment with MTX) |
| Adalimumab | 2002 (RA,FDA) | CD and UC (pediatric and adult) JIA, RA, SpA, AS, nr-axSpa, Uveitis , (pediatric and adult), HS, Ps (adult and pediatric) | RA,AS,PsA,axSpA: sc, 40 mg every 2-weeks; Ps:sc, 80mg at 0, then 40mg every 2 weeks CD/UC: sc, 80-160mg at week 0, 40-80mg at week 2, then 40mg every 2 weeks | Pediatric IBD, Uveitis, HS |
| Certolizumab pegol | 2008 (CD,FDA) | RA, AS, nr-axSpa, PsA, Ps (adult) | RA,PsA,axSpA,Ps: sc, 400 mg at 0, 2, 4-weeks, then 200-400 mg every 2 weeks (or 400 mg monthly) | No pediatric indications; low placental transfer |
| Golimumab | 2009 (RA, FDA) | RA, SpA, JIA, AS, PsA, nr-axSpa, UC | RA,PsA,AS,nr-axSpA: sc, 50 mg every 4-weeks; UC: sc, 200 mg at week 0, 100mg at week 2, then 50-100 mg every 4 weeks | |

Abbreviations: mAb, Monoclonal Antibody; RA, Rheumatoid Arthritis; SpA, Spondyloarthritis, including nr-axSpA; PsA, Psoriatic Arthritis; JIA, Juvenile Idiopathic Arthritis; CD,Crohn's Disease; UC, Ulcerative Colitis; HS, hidradenitis suppurativa; AS, ankylosing spondylitis; iv, Intravenous Infusion; sc, Subcutaneous Injection; PEG, Pegylated; TNFR, Tumor Necrosis Factor Receptor. *Indications, administration route and dosing are extracts of the respective currently available SmPCs. For simplification, only the rheumatologic and gastroenterologic indications were listed excluding pediatric indications. For exact dosing instructions for children, as well as for the other approved indications such as uveitis or HS, please refer to the currently locally approved SmPC.
